# Proteins and lipids of glycosomal membranes from *Leishmania tarentolae* and *Trypanosoma brucei*

**DOI:** 10.12688/f1000research.2-27.v1

**Published:** 2013-01-29

**Authors:** Claudia Colasante, Frank Voncken, Theresa Manful, Thomas Ruppert, Aloysius G M Tielens, Jaap J van Hellemond, Christine Clayton

**Affiliations:** 1 Institut für Anatomie und Zellbiologie, Giessen, 35392, Germany; 2 Department of Biological Sciences and Hull York Medical School, University of Hull, Hull, HU6 7RX, UK; 3 Department of Biochemistry, Cell & Molecular Biology, University of Ghana, Accra, P.O. Box LG 54, Ghana; 4 DKFZ-ZMBH Alliance, Zentrum für Molekulare Biologie der Universität Heidelberg, Heidelberg, D69120, Germany; 5 Department of Medical Microbiology and Infectious Diseases, ErasmusMC University Medical Center, Rotterdam, PO box 2040, Netherlands; 6 Department of Biochemistry and Cell Biology, Faculty of Veterinary Medicine, Utrecht University, Utrecht, PO Box 80176, Netherlands

## Abstract

In kinetoplastid protists, several metabolic pathways, including glycolysis and purine salvage, are located in glycosomes, which are microbodies that are evolutionarily related to peroxisomes. With the exception of some potential transporters for fatty acids, and one member of the mitochondrial carrier protein family, proteins that transport metabolites across the glycosomal membrane have yet to be identified. We show here that the phosphatidylcholine species composition of *Trypanosoma brucei* glycosomal membranes resembles that of other cellular membranes, which means that glycosomal membranes are expected to be impermeable to small hydrophilic molecules unless transport is facilitated by specialized membrane proteins. Further, we identified 464 proteins in a glycosomal membrane preparation from *Leishmania tarentolae*. The proteins included approximately 40 glycosomal matrix proteins, and homologues of peroxisomal membrane proteins - PEX11, GIM5A and GIM5B; PXMP4, PEX2 and PEX16 - as well as the transporters GAT1 and GAT3. There were 27 other proteins that could not be unambiguously assigned to other compartments, and that had predicted trans-membrane domains. However, no clear candidates for transport of the major substrates and intermediates of energy metabolism were found. We suggest that, instead, these metabolites are transported via pores formed by the known glycosomal membrane proteins.

## 1. Introduction

In kinetoplastid protists, several metabolic pathways, including glycolysis, purine salvage and ether lipid biosynthesis, are located in a microbody, the glycosome ^1, 2^, which is evolutionarily related to peroxisomes. All evidence so far indicates that the glycosomal membrane, like the peroxisomal membrane, is impermeable to nucleotides, notably adenosine phosphates and NAD(P)(H) ^3, 4^. Its permeability to smaller molecules, however, is subject to debate ^5^. Specific transporters would be required if the membrane were impermeable to molecules of the size of glycolytic intermediates, such as glucose, phosphate, malate, pyruvate, phosphoenolpyruvate and various triosephosphates.

In 1987, the first protein profile of glycosomal membranes from *Trypanosoma brucei* was published ^6^. It revealed two abundant proteins of 24 and 26 kDa, which were later shown to be trypanosome homologues of the peroxisome biogenesis protein PEX11 ^7– 9^. Subsequent studies, including two of the glycosomal proteome ^1, 10^, revealed several more trypanosome PEX proteins that are predicted to be membrane-bound, such as PEX2 ^11, 12^, PEX10 ^13^, PEX12 ^13^, PEX13 ^14^ and PEX14 ^15^. The only transporters known to be associated with the glycosomal membrane are the ABC transporters GAT1, GAT2 and GAT3, which might transport fatty acids ^16^. In addition, a member of the mitochondrial carrier protein family was found: MCP6, which is a candidate for nucleotide transport ^17^. MCP6 is found preferentially in the glycosomal membranes of bloodstream-form trypanosomes, whereas in procyclic forms, it is predominantly targeted to the mitochondria ^17^.

No analysis has yet yielded evidence for glycosomal transporters of metabolites smaller than about 400 Da. In contrast, lipid bilayers that were reconstituted with glycosomal membrane proteins revealed evidence for the presence of anion- and cation-selective pores ^5^. The identities of these pore-forming proteins are still unknown: they could be dedicated exclusively to metabolite transport, or they might be involved in protein import as well ^5^.

If proteins other than PEX components were indeed involved in metabolite transport, it ought to be possible to find them by mass spectrometry, using highly purified glycosomal membrane protein preparations. A similar proteomics approach has been previously used for mammalian peroxisomes. Analysis of carbonate-washed rat liver peroxisomes initially yielded only two peroxisomal membrane proteins, PMP70 and PMP22 ^18^, whereas a later analysis of whole mouse kidney peroxisomes led to the identification of 12 putative glycosomal membrane proteins. These included one tetratricopeptide domain protein, four different ABC transporters, three members of the PMP22 family, PMP34, Pxmp4/PMP4, and the putative solute carrier PMP47 ^19^.

Specific transporters for glycolytic metabolites might have been missed in previous glycosomal proteomic analyses, since glycosomal membrane proteins are likely to comprise a rather small proportion of the total protein content. We have therefore set out to identify the proteins in a highly enriched glycosomal membrane preparation from *Leishmania tarentolae*, using 30-times more starting material than used for our previously published *T. brucei* glycosomal proteome study ^1^. Comprehensive mass spectrometry analysis of these highly purified glycosomal membrane protein fractions did not, however, lead to the identification of any novel glycosomal transporters. We therefore postulate that the recently described porin activity ^5^ in the glycosomal membrane might be provided by known components of the glycosomal protein import machinery (peroxins), as has also been suggested for peroxisomes ^20^. In addition, we compared the phospholipid compositions of the glycosomal membranes from bloodstream- and procyclic-form *T. brucei*, with the lipid composition of the *T. brucei* cell membrane to see if this could give us more information regarding glycosomal membrane transport.

## 2. Materials and methods

### 2.1. Isolation of glycosomes from *Trypanosoma brucei* and *Leishmania tarentolae*

*Leishmania tarentolae* promastigotes were cultured at 28°C in 3.7 L hemin-supplemented brain-heart infusion medium to a maximum density of 2 × 10 ^8^ cells/ml. Procyclic-form *Trypanosoma brucei* Lister 427 was grown at 30°C in 10% (v/v) foetal calf serum-supplemented MEM-PROS medium to a maximum density of 5 × 10 ^6^ cells/ml ^21^. Bloodstream-form *T. brucei* 427 was grown at 37°C in 10% (v/v) foetal calf serum-supplemented HMI-9 medium ^22^ to a maximum density of 2 × 10 ^6^ cells/ml ^21, 23^.

Procyclic-form and bloodstream-form *T. brucei* (10 ^10^ cells each), and promastigote *L. tarentolae* (10 ^12^ cells) were harvested by centrifugation for 10 min at 2,000x g, and were washed once in 50 ml of TEDS (25 mM Tris, 1 mM EDTA, 1 mM DTT, 250 mM sucrose, pH 7.8). After centrifugation, the cell pellet was resuspended in 2 ml homogenization medium (250 mM sucrose, 1 mM EDTA, 0.1% (v/v) ethanol, 5 mM MOPS, pH 7.2) containing protease inhibitor (complete EDTA-free, Roche Applied Science) and was grinded in a pre-chilled mortar with 1 volume of wet-weight silicon carbide (Crysalon: Norton Company: porous <400 mesh). Cells were checked for at least 90% disruption by light microscopy. The cell lysate was centrifuged sequentially for 5 minutes each at 100x g and 3,000x g to remove abrasive, intact cells, cell rests and nuclei. The supernatant was centrifuged for 15 minutes at 17,000x g to yield the glycosome-enriched pellet fraction. This fraction was resuspended in 3 ml of homogenization buffer and loaded on top of a 32 ml linear 20–40% (v/v) Optiprep (iodixanol-sucrose, Sigma Biochemicals) gradient, mounted on a 3.5 ml 50% (v/v) Optiprep cushion (Optiprep Application Sheet S9, Axis-shield). Centrifugation was performed for 1 h at 170,000x g and 4°C using a Beckman VTi-50 Rotor. 1 ml aliquots were collected from the bottom of the tube after puncture, and the protein concentration of each fraction was determined using the BioRad Bradford protein assay. Of each fraction, 100 µl was TCA-precipitated and the resulting pellets resuspended in denaturing Laemmli SDS-PAGE buffer. Proteins were separated on a 12% SDS-PAGE gel and analysed by western blotting.

### 2.2. Isolation and analysis of glycosomal membrane proteins from *Leishmania tarentolae* glycosomes

Glycosomes (corresponding to about 0.5 mg protein) were diluted 1:5 in TEDS (see 2.1), subjected to two freeze-thaw cycles, and centrifuged for 40 minutes at 140,000x g and 4°C. The resulting pellet was washed with 5 M urea for 1 h at 4°C to remove proteins that were not tightly associated with the glycosomal membranes. The glycosomal membranes were pelleted by centrifugation for 40 minutes at 140,000x g and 4°C. This 5 M urea wash-step was repeated once. The glycosomal membrane-enriched fraction was resuspended in denaturing Laemmli SDS-PAGE buffer and separated by SDS PAGE. In-gel trypsin digestion and nanoLC-MS/MS analysis of the obtained protein bands were performed as previously described ^1^. The obtained MS/MS spectra were analysed using MASCOT software and visualised in Scaffold. The comparison shown in Supplementary Table 1 was done using the 2012 version of the shotgun sequence of *L. tarentolae* ( http://tritrypdb.org/) ^24^; only proteins for which at least 2 different peptides could be identified with >95% confidence were included. *Leishmania* proteins were first scanned for the presence of a PTS1 signal based on a published analysis for *L. major* and *T. brucei* ^2^ and by manually examining the C-terminal sequences. Additional PTS1-containing proteins were identified using PTS1 Predictor ^25, 26^. Trans-membrane domains were identified using the TritrypDB annotation database. For potential glycosomal proteins with no known function and without an annotated trans-membrane domain, we also scanned for trans-membrane domains using the HMMTOP and SOSUI algorithms ^27, 28^.

### 2.3. Phospholipid analysis

Lipids were extracted in triplicate from bloodstream and procyclic *T. brucei* samples and from a single batch of isolated glycosomes due to the limited amount of purified material. Lipids were extracted according to the method of Bligh and Dyer (1959) ^29^ with the minor modification that 0.5% (v/v) 6 M HCl was added to the second chloroform wash to increase recovery of acidic phospholipids. The phospholipids and free fatty acids were separated from neutral lipids (cholesterol, cholesterol esters and triacylglycerols) by fractionation on a 1 ml silica column prepared from 0.063–0.200 mm silica 60 (Merck, Darmstadt, Germany). Lipid extracts were dissolved in chloroform and loaded on the silica column, then eluted successively with acetone (4 volumes) and methanol (4 volumes). The last fraction, which contained the purified phospholipids, was dried under nitrogen and stored at -20°C until HPLC-MS analysis.

The purified phospholipids were dissolved in methanol:acetonitrile:chloroform:water (46:20:17:17). Separation of molecular lipid species was performed on a Synergi 4 µm MAX-RP 18A column (250 × 3 mm; Phenomenex, CA, USA). Elution was performed with a linear gradient of water in methanol/acetonitrile (60/40 (v/v)) decreasing from 12.5% to 0% in 25 min, followed by further isocratic elution for another 25 minutes. The flow rate was kept constant at 0.425 ml•min ^-1^ and 1 µM serine and 2.5 mM ammonium acetate were used in all solvents as additives.

Mass spectrometry of lipids was performed using electrospray ionization, on a 4000 QTRAP system (Applied Biosystems, Nieuwerkerk aan de IJssel, The Netherlands). Source temperature was set to 450°C and nitrogen was used as curtain gas. The declustering potential was optimized using lipid standards. The optimal collision energy was dependent on the type of experiment performed and was set to +45V (precursor scanning m/z 184), -45V (precursor scanning m/z -196), +35V (neutral loss 141), -30V (precursor scanning m/z -241), and -40V (neutral loss scanning 87 Da) respectively. For quantification of molecular species, samples were measured in multiple-reaction monitoring mode (MRM), monitoring for 95 head-group specific mass transitions with a total dwell time of 1 s, using the same settings as above. Data analysis was performed with Analyst™ v 1.4.1 software (MDS Sciex, Concord, ON).

Supplementary Table S1 All proteins, possible glycosomal proteins, and a short list of glycosome membrane proteins.

## 3. Results

### 3.1. Glycosome preparation from *Leishmania tarentolae*

**Figure 1.**
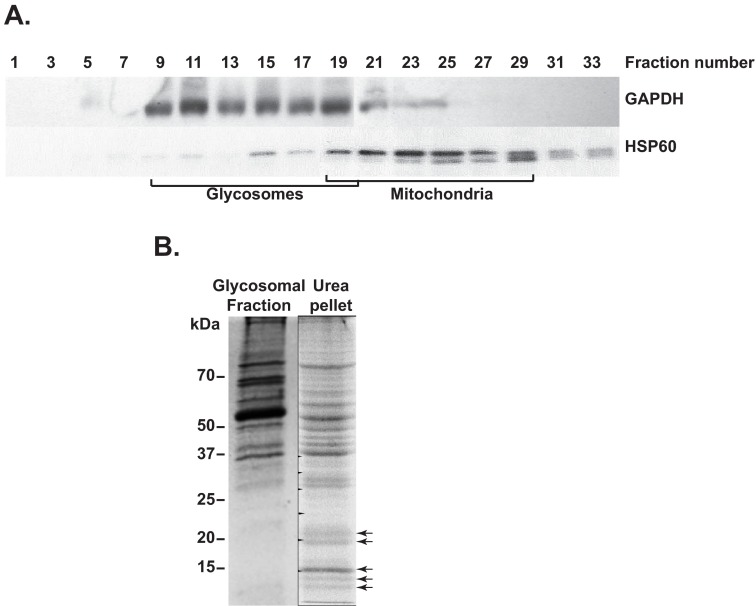
Purification of glycosomal membrane-enriched fractions from *L. tarentolae*. **A**. Western blot analysis of the different *L. tarentolae* fractions obtained after density gradient centrifugation. Equal volumes of only the odd-numbered fractions were loaded for analysis. Antibodies used for detection are indicated next to the western blot panels. Mitochondrial ^19– 29^ and glycosomal ^9– 19^ density gradient fractions are indicated. **B**. SDS-PAGE gel stained with Coomassie brilliant blue, showing protein bands from intact glycosomes (glycosomal fraction, and from the glycosomal membrane-enriched pellet (urea pellet). Arrows indicate enriched proteins in the urea-treated glycosomal membrane fraction.

In preliminary experiments (not shown), we tested various methods for purification of membranes of iodixanol gradient-enriched *T. brucei* glycosomes ^1^. The methods tested included methanol/chloroform extraction ^30^; ultracentrifugation of glycosomes that had been subjected to osmotic shock with cold water ^31, 32^; and different high salt (0–1 M NaCl or 5 M urea) washes of glycosomal membranes ^32^. Although we were able to enrich glycosomal membrane proteins, as judged by the presence of PEX11 ^7^, the matrix protein aldolase persisted. In addition, the total amount of membrane protein obtained from 3 × 10 ^10^ *T. brucei* was so low that we doubted that any lower-abundance proteins would be detected by mass spectrometry. We therefore decided to isolate glycosomes from the related kinetoplastid *L. tarentolae* to increase the sensitivity for the detection of even low-abundant glycosomal membrane proteins. In contrast to *T. brucei, L. tarentolae* can be grown to far higher cell densities, enabling us to isolate glycosomes from as much as 10 ^12^ cells. The different fractions obtained after differential fractionation and subsequent density gradient (Optiprep) centrifugation were analysed by western blotting ( Figure 1). The gradient distributions of the two marker proteins glyceraldehyde phosphate dehydrogenase (glycosomes) and HSP60 (mitochondria) are shown in Figure 1A. Comparison of previously published density gradient results from *T. brucei* ^1^ with those from *L. tarentolae* ( Figure 1A) showed that the mitochondria were enriched at similar gradient densities (fractions 22–25), whereas the glycosomes isolated from *L. tarentolae* appeared to have a higher buoyant density than those of *T. brucei*. In addition, both the mitochondria and glycosome-containing fractions were spread out over a wide range of fractions for the *L. tarentolae* gradient, which could be the result of breakage of the organelles during isolation. Judging from the western blotting results (alternate gradient fractions shown in Figure 1A), fractions 9, 11 and 13 contained about 42% of the total GAPDH measured, but only 2% of the total HSP60. We therefore decided to use fractions 9–13 for further glycosomal membrane purification.

To purify glycosomal membranes, we found that the protocol that gave least matrix protein contamination was one that was successfully employed to isolate the cell membrane of *E. coli* ^33^. It involved washing the glycosomal pellet with 5 M urea, and resulted in strong depletion of some prominent bands (presumably matrix proteins) and the enrichment of various proteins in the 10–25 kDa range ( Figure 1B) - similar to the expected sizes of the PEX11 protein homologues ^7, 9^. The entire SDS-PAGE lane containing the enriched glycosomal protein fraction ( Figure 1B) was subsequently subjected to mass spectrometry.

### 3.2. Identification of putative glycosomal proteins

**Supplementary Figure S1.**
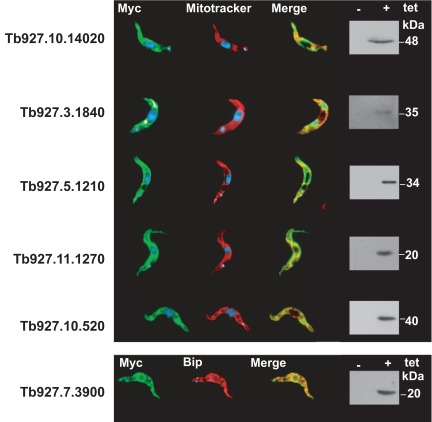
Immunofluorescence analysis of the putative glycosomal membrane proteins in procyclic *T. brucei*. N-terminally or C-terminally myc-tagged versions of the proteins were detected (green) by using a commercial Myc antibody (Sigma-Aldrich). Mitochondria were visualized (red) using mitotracker. The endoplasmatic reticulum (ER) was detected using an antibody directed against the ER lumen protein BiP (red). Overlays (Merge) of the green staining and the red staining are shown to visualize the common compartmentalization of the proteins. On the right side, western blots are shown to illustrate the expression of the myc-tagged membrane proteins. (+) and (-) indicate tetracycline-induced and -uninduced cells respectively.

By comparison with the predicted proteome of *L. tarentolae* ^24^, 464 polypeptides were identified ( Supplementary Table S1). The first step that we undertook was to identify homologues of all identified proteins from the *T. brucei* genome ( http://tritrypdb.org/tritrypdb/). This was done to facilitate the retrieval of information because most experimental data is available exclusively for *T. brucei.* All identified proteins were screened for database annotation, including user comments, and in some cases we also updated annotations from publications. We further screened all proteins for their presence in previously published glycosomal ^1, 10^ and mitochondrial ^34^ proteomes. The results are summarised in Supplementary Table S1, Sheet 1. Proteins that were clearly located in compartments other than the glycosome were then excluded, resulting in Supplementary Table S1, Sheet 2. Some candidates predicted to contain at least one trans-membrane domain were tested for their locations, by expression of N-terminally and/or C-terminally tagged versions (none has a PTS1 signal). The proteins encoded by Tb927.3.1840 (putative 3-oxo-5-alpha-steroid 4-dehydrogenase), Tb927.5.1210 (putative short-chain dehydrogenase) and Tb927.10.14020 (unknown function) were all targeted to mitochondria, while Tb927.7.3900 (annotated as a vacuolar transporter chaperone) was in the ER (Supplementary Figure S1). All identified proteins were further searched for the presence of known peroxisomal targeting signals in the *L. major* or *T. brucei* homologues ^35^; in addition, the *L. tarentolae* protein sequences in Supplementary Table S1, Sheet 2 were manually scanned for PTS1 signals.

### 3.3. Glycosomal enzymes

**Figure 2.**
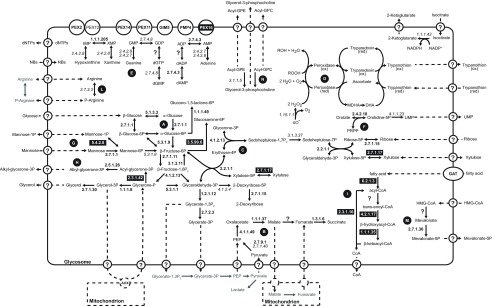
Putative glycosomal pathway scheme. The scheme summarizes metabolic pathways identified so far in the glycosomes of *Leishmania* and *T. brucei*. EC numbers of enzymes identified only in the *Leishmania* glycosome are indicated in black boxes with white text, those identified only in *T. brucei* are indicated in italics, and those found in the glycosome of both species are indicated in bold. Predicted transport processes across the glycosomal membrane are indicated by the circled question marks and dashed arrows. Letters in black circles indicate the different metabolic pathways as follows: A: glycolysis; B: succinic fermentation; C: pentose phosphate pathway; D: superoxide and trypanothione metabolism; E: purine salvage; F: pyrimidine metabolism; G: mannose metabolism; H: glycerolipid biosynthesis; I: β-oxidation of fatty acids; L: phosphoarginine metabolism; M: mevalonate pathway; N: phospholipid degradation. Abbreviations used are: Acyl-GPC, 1-acyl-glycero-phosphocholine; Acyl-GPE, 1-acyl-glycero-phosphoethanolamine; AOX, alternative oxidase; DHA, dehydroascorbate; MDHA, monodehydroxyascorbate; NB, nucleobases; PRPP, 5-phosphoribosyl 1-pyrophosphate.

The *L. tarentolae* glycosomal membrane preparations revealed the presence of 40 known or predicted glycosomal matrix proteins, and some novel proteins ( Supplementary Table S1, sheet 2). A putative glycosomal pathway scheme, incorporating all available information for *L. tarentolae* and *T. brucei*, is shown in Figure 2. Glycolytic enzymes, enzymes involved in the conversion of glycerone phosphate to glycerol, the pentose phosphate pathway, steroid and nucleotide biosynthesis as well as enzymes of the succinic fermentation branch were detected. Similar to results obtained for the *T. brucei* glycosome ^1^, fumarase (EC 4.2.1.2) is the only enzyme of the glycosomal succinic fermentation branch that was not found in the *L. tarentolae* glycosomal membrane preparation. It is possible that fumarase was removed in the membrane purification; alternatively, the activity may be supplied by one of the four proteins of unknown function that are conserved in kinetoplastids and have an unambiguous PTS1: LtaP34.3290/Tb927.4.1360, LtaP33.2650/Tb927.11.2620, LtaP18.0870/Tb927.10.13240, or LtaP24.1780/Tb927.8.6640 - although fumarase activity would be surprising since they lack known functional domains. Fumarase catalyses the conversion of malate to fumarate, which is an indispensable step towards the generation of succinate in the glycosomal matrix. If indeed the glycosome lacks fumarase then the glycosomal membrane must harbour a malate-fumarate shuttle. This shuttle would be responsible for the transport of glycosome-derived malate in exchange with mitochondria-derived fumarate. Inside the glycosome fumarate could then be converted to succinate by fumarate reductase (EC 1.3.1.6) to maintain the glycosomal NADH balance.

The *L. tarentolae* glycosomal membrane preparation contained several enzymes that were not detected during LC-MS analysis of the *T. brucei* glycosome. For example, glucosamine-6-phosphate isomerase was found in the glycosomes of *Leishmania*, but not in trypanosomes ^1, 10^. In addition, a PTS1-containing D-lactate dehydrogenase-like protein is found in *Leishmania*, for which there is no obvious substrate, as well as a PTS1-containing xylulokinase and a glucokinase-like protein. These additional enzymes involved in the metabolism of sugars might indicate a higher metabolic flexibility of *L. tarentolae* compared to African trypanosomes. In *T. brucei*, the phosphomannomutase Tb927.10.6440 was found in the glycosome, where it can act as phosphoglucomutase during glycolysis ^36^. *T. brucei* phospho-N-acetylglucosamine mutase (Tb927.8.980) was also partially glycosomal ^36^. Neither has an obvious PTS1 targeting signal so it is possible that they have either an internal glycosomal targeting signal or that they are co-imported via association with other glycosomal targeting signal-containing proteins ^37, 38^. The syntenic *L. tarentolae* homologues LtaP36.1960 and LtaP07.0850 were not present in our dataset, but the different non-syntenic phosphomannomutase-like protein LtaP34.3710, containing the conserved C-terminal PTS1 signal -SKL, was found. The *T. brucei* aldose 1-epimerase, Tb927.4.1360, is the homologue of LtaP34.3290, but Leishmanias have an additional isoform, LtaP35.110, containing a conserved PTS1 targeting signal.

The first three steps of ether-lipid biosynthesis in *T. brucei*, namely the conversion of glycerone 3-phosphate to 1-alkyl-glycerol-3-phosphate, are associated with the glycosome ^39^. *T. brucei* glycosome proteomic analysis confirmed the presence of the second enzyme of the biosynthetic pathway, namely alkyl-glycerone-phosphate synthetase (EC 2.5.1.26). The first and the third enzymes, glycerone-phosphate acyl-transferase (EC 2.3.1.42) and 1-acyl-glycerol-3-phosphate oxidoreductase (EC 1.1.1.101) were not identified. The *T. brucei* glycerone 3-phosphate acetyltransferase/acyltransferase homologue, Tb927.4.3160, has a C-terminal PTS1, SRM. Analysis of the glycosomal membrane proteome of *L. tarantolae* identified not only alkyl-glycerone 3-phosphate synthetase (EC 2.5.1.26) but also glycerone 3-phosphate acetyltransferase/acyltransferase (EC 2.3.1.42, LtaP34.1280, C-terminal PTS1 -SKM) supporting the idea that the first two steps of the ether-lipid biosynthesis can occur inside the glycosome.

The β-oxidation of long chain fatty acids (LCFA) is one of the hallmark catabolic pathways attributed to peroxisomes ( ^40^ and references therein). Our previous analysis suggested that the glycosome of *T. brucei* was devoid of LCFA β-oxidation enzymes ^1^. On the other hand, 2-enoyl coenzyme A hydratase (EC 4.2.1.17) and NADP-dependent 3-hydroxyacyl-CoA dehydrogenase (EC 1.1.1.35) were reported in glycosomal fractions from procyclic-form *T. brucei* ^40^. According to the results from this proteome analysis, the *L. tarentolae* glycosome might contain all enzymes involved in the β-oxidation of LCFA except acyl-CoA dehydrogenase ( Figure 2, Supplementary Table S1, sheet 2). We found one protein (LtaP24.1780, annotated as hypothetical), which is clearly a fatty acyl Co-A reductase and contains a C-terminal -SSL; another hypothetical protein (LtaP16.0130) contains -AKL and an acyl CoA dehydrogenase domain; and the enoyl-CoA hydratase/enoyl-CoA isomerase/3-hydroxyacyl-CoA dehydrogenase trifunctional enzyme homologues (EC 4.2.1.17/5.3.3.8/1.1.1.35, LtaP26.1590 and LtaP33.2830) containing putative PTS2 signals ^35^. Other detected enzymes of this pathway without obvious peroxisomal targeting signals were long-chain-fatty-acyl-CoA synthetase (EC 6.2.1.3) and thiolase (EC 2.3.1.16); these might have internal signals or could be contaminants from another compartment.

Mevalonate kinase is known to be in the glycosome ^41^, and another enzyme of the mevalonate pathway, isopentenyl-diphosphate delta-isomerase, was also present in our glycosomal preparation; but intermediate enzymes (phosphomevalonate kinase and mevalonate-5-pyrophosphate decarboxylase) were not ( Figure 2, Supplementary Table S1, sheet 2). Finally, we found a PTS1-containing phosphoribulokinase/uridine kinase family protein, LtaP14.0950, belonging to the P-loop NTPase superfamily; we speculate that this enzyme might be involved in pyrimidine salvage.

### 3.4. Putative glycosomal membrane proteins

We next focussed on the identification of putative membrane proteins. Known glycosomal membrane proteins and unassigned proteins that had predicted membrane-spanning domains in both *L. tarentolae* and *T. brucei* are listed in Supplementary Table S1, sheet 3. These proteins were manually analysed for conserved functional domains, and their protein sequences were aligned to the complete predicted proteomes of *Homo sapiens*, *Saccharomyces cerevisiae*, *Hansenula polymorpha* and *Pichia pastoris* on the NCBI Web site using Blastp. This enabled the exclusion of further proteins that were deemed likely to be in other (non-peroxisomal) subcellular compartments.

The list of known glycosomal membrane proteins that we identified is shown in Table 1. It included three proteins related to PEX11 (PEX11, GIM5A and GIM5B), PEX2 and PEX14. The protein encoded by Tb09.160.4700/Tb927.9.6450 has a very weak match to a conserved PEX16 domain (E-value 9e ^-3^). We have therefore annotated this as a putative PEX16. It could therefore be involved in the incorporation of peroxisomal membrane proteins ^42^, although high-throughput RNAi screening revealed no growth defects for RNAi targeting this locus ^43^.

**Table 1.** Known proteins of the glycosomal membrane. This list includes all mass-spectrometry-detected glycosomal membrane proteins.

Lta gene ID	Peptides	Coverage	Tb gene ID	Function
LtaP28.2340	16	59%	Tb927.11.11520	PEX11
LtaP24.0140	7	25%		
LtaP28.2330	3	14%		
LtaP25.2350	3	8%	Tb927.3.2340	PEX2
LtaP15.0800	6	13%	Tb927.9.6450	PEX16
LtaP28.1050	3	15%	Tb11.0300, Tb11.0400	
LtaP26.2550	2	13%	Tb927.9.1720	PMP4
LtaP35.3750	12	48%	Tb927.9.11580, Tb927.9.11600	GIM5A,B
LtaP31.0560	5	5%	Tb927.11.3130, Tb927.4.4050	GAT2
LtaP27.0470	4	6%	Tb927.11.1070	GAT3

Abbreviations used: Lta, *Leishmania tarentolae*; Tb, *Trypanosoma brucei*.

The trypanosome homologue of the mammalian peroxisomal membrane protein PMP24 ^44^ (also called PXMP4 or PMP4) was also found. Like other PMP24/PMP4 proteins, a conserved TIM27 superfamily domain is present in Tb927.9.1720; the function of this domain (and of PMP4) is still unknown. Although *S. cerevisiae* that lack it show abnormal peroxisome size and numbers ^45^, no growth defects were seen in the high-throughput RNAi screens in *T. brucei* ^43^. Of the previously reported *T. brucei* glycosomal ABC transporters GAT1-3 ^16^, only GAT1 and GAT3 were found in the *L. tarentolae* glycosomal proteome. This was not unexpected since the annotated *L. tarentolae* GAT2 protein sequence is severely truncated. Either *L. tarentolae* lacks a functional GAT2, or this is a genome assembly error; the former is quite possible since no peptides matching *L. major* and *L. infantum* GAT2 were found.

Of the remaining potential membrane proteins, four could be tentatively assigned to the mitochondrion, three to the endoplasmic reticulum, two to the flagellum, and one to the nucleus ( Supplementary Table S1, sheet 3). The remaining proteins are listed in Table 2. A protein of the major facilitator family (Tb927.3.4070-110, LtaP29.1650) was the only conserved multi-pass membrane protein that had no clear assignment to another subcellular compartment, but the 3 identified peptides covered only 3% of the protein. Best matches to this sequence are ion transporters. The Tb927.9.4310 protein sequence matches a yeast possible alpha-isopropylmalate carrier, which exports alpha-isopropylmalate from the mitochondrion to the cytoplasm for use in leucine biosynthesis. The remaining candidates have either one or two potential trans-membrane domains, usually predicted by only one algorithm.

**Table 2.** Possible additional glycosomal membrane proteins. This list includes all detected proteins with predicted trans-membrane (TM) domains, for which there is no evidence for location in any particular compartment.

Lta gene ID	Pep nr	Cov (%)	Tb gene ID	Function	TMs (a)	TMs (b)	ER sp
LtaP29.1650	3	3	Tb927.3.4070 Tb927.3.4110	Major facilitator family, nitrate and chloride transporter?	12	nd	no
LtaP01.0560	4	17	Tb927.9.4310	putative tricarboxylate carrier, matches yeast putative alpha-isopropylmalate carrier, which exports alpha-isopropylmalate from the mitochondrion to the cytoplasm for use in leucine biosynthesis	5	nd	no
LtaP05.0670	2	6	Tb927.9.4310	putative tricarboxylate carrier, matches yeast putative alpha-isopropylmalate carrier, which exports alpha-isopropylmalate from the mitochondrion to the cytoplasm for use in leucine biosynthesis	5	nd	no
LtaP36.4130	3	6	Tb927.11.10260	hypothetical protein, conserved	0	2	no
LtaP33.2680	4	4	Tb927.11.2590	hypothetical protein, conserved	0	2	no
LtaP29.0320	2	21	Tb927.3.5350	hypothetical protein, conserved	1	1	no
LtaP36.6980	4	4	Tb927.10.8000	P-loop nucleoside triphosphate hydrolase, contains EF hand domains	0	1	no
LtaP21.0440	4	6	Tb927.10.2240	NTF2-like domain	0	1	no
LtaP11.1210	2	5	Tb927.11.6070	ARM repeat superfamily	0	1	no
LtaP31.3020	2	3	Tb927.4.5000, Tb927.8.7420	TERD-like domains	0	1	yes
LtaP35.4380	2	4	Tb927.9.10470	hypothetical protein, conserved	0	1	no
LtaP34.2250	2	5	Tb927.4.2250	hypothetical protein, conserved	0	1	no
LtaP06.1080	3	11	Tb927.7.5700	hypothetical protein, conserved	1	nd	yes
LtaP26.0630	3	12	Tb927.7.1290	hypothetical protein, DUF2012, peptidase superfamily, starch binding domain, similarity to human C15ORF24	1	nd	yes
LtaP36.3480	2	8	Tb927.11.10040	hypothetical protein, conserved	1	nd	no
LtaP17.1410	2	36	Tb927.5.2560	hypothetical protein, conserved	1	nd	no
LtaP23.0390	3	10	Tb927.8.2300	hypothetical protein, conserved	1	nd	no

Abbreviations used: Lta, *Leishmania tarentolae*; Tb, *Trypanosoma brucei*; Pep nr, number of identified peptides; Cov (%), percentage of protein coverage; TMs (a), number of annotated trans-membrane domains; TMs (b), number of transmembrane-domains identified by THMM TOP; ER sp, endoplasmic reticulum signal peptide.

Overall, our proteomics analysis revealed no clear candidates for major novel glycosomal metabolite transporters.

### 3.5. Phosphatidylcholine composition of cellular and glycosomal membranes

The phosphatidylcholine species composition in membranes from total *T. brucei* cells and glycosomes were analysed to detect possible differences in membrane composition between glycosomal and the other membranes of *T. brucei*, and to allow comparison in membrane composition between the two cultivatable replicating life cycle stages, the bloodstream form and procylic form. The phosphatidylcholine species composition of total trypanosome membranes differed to some extent between bloodstream and procyclic stages ( Table 3), which is consistent with earlier reports on the phospholipid composition in *T. brucei* ^46^. Membranes of both stages contain predominantly diacyl-phosphatidylcholine species, comprising common fatty acids consisting of 16 to 22 carbon atoms with 0 to 6 desaturations. In addition, membranes of intact trypanosomes also contained ether phospholipids species, both 1-alkyl-2-alkyl phosphatidylcholine species and 1-alkyl-2-acyl phosphatidylcholine species. The phosphatidylcholine species composition of glycosomal membranes differed only to a minor extent from the composition observed in total membranes of both bloodstream form and procyclic form trypanosomes ( Table 3).

**Table 3.** Phosphatidylcholine composition of procyclic-form and bloodstream-form *T. brucei* total cells and glycosomes.

			PCF		BSF	
Peak	Component		Glycosomes	Total cells	Glycosomes	Total cells
			Mol %	Mol %	Mol %	Mol %
1	PtdCho	16:0, 16:1	2,9	1,7 ± 0,0	0,5	0,2 ± 0,2
2	AlkCho	16:0, 18:2	2,3	2,1 ± 0,3	0,7	nd
3	PtdCho	16:0, 18:3	3,3	1,7 ± 0,3	0,5	nd
4	PtdCho	16:0, 18:2	**7,9**	3,9 ± 0,5	2,5	3,9 ± 0,6
5	PtdCho	16:0, 18:1	2,8	3,1 ± 0,9	3,2	1,2 ± 0,3
6	AlkCho	16:0, 20:3	1,0	0,6 ± 0,0	0,2	nd
7	EnylCho	18:0, 18:2	0,9	1,0 ± 0,2	1,4	1,0 ± 0,2
8	AlkCho	18:0, 18:2	**9,3**	**12,8 ± 0,7**	**9,6**	**6,6 ± 0,3**
9	PtdCho	18:2, 18:2, 18:1, 18:3	**12,6**	**6,1 ± 0,5**	**5,1**	4,4 ± 0,4
10	PtdCho	18:1, 18:2	**8,1**	**6,8 ± 0,2**	4,3	**5,6 ± 0,7**
11	PtdCho	18:0, 18:2, 18:1, 18:1	**23,2**	**13,4 ± 0,7**	**26,0**	**32,7± 4,6**
12	PtdCho	18:0, 18:1	2,4	1,2 ± 0,2	2,4	2,2 ± 0,4
13	AlkCho	16:0, 22:1, 18:0, 20:1	0,5	0,6 ± 0,1	3,5	0,4 ± 0,7
14	PtdCho	16:0, 22:6	1,4	0,9 ± 0,1	nd	nd
15	PtdCho	18:1, 20:5	2,8	3,9 ± 0,3	2,3	3,7 ± 0,3
16	PtdCho	16:0, 22:5	1,1	0,8 ± 0,0	1,1	2,1 ± 0,6
17	PtdCho	18:1, 20:4	1,3	2,6 ± 0,1	nd	nd
18	PtdCho	16:0, 22:4	0,9	0,8 ± 0,2	2,3	2,2 ± 0,6
19	PtdCho	18:0, 20:3	2,2	1,8 ± 0,7	3,0	4,1 ± 0,4
20	PtdCho	18:3, 22:5	4,0	**12,2 ± 0,1**	4,9	4,5 ± 0,2
21	PtdCho	18:2, 22:5	0,3	nd	2,4	1,7 ± 0,3
22	PtdCho	18:1, 22:6	3,3	**9,5 ± 2,1**	3,5	3,5 ± 0,7
23	PtdCho	18:3, 22:3	0,4	0,9 ± 0,3	3,0	2,6 ± 1,0
24	PtdCho	18:0, 22:6	2,4	3,2 ± 0,3	3,2	**5,3 ± 0,8**
25	PtdCho	18:0, 22:5	2,6	**5,5 ± 0,4**	3,7	3,6 ± 0,3
26	PtdCho	18:2, 22:3	0,0	nd	3,2	3,4 ± 0,3
27	PtdCho	18:0, 22:4	0,3	0,5 ± 0,1	**6,7**	3,7 ± 0,4
28	PtdCho	18:0, 22:3	0,2	0,2 ± 0,1	0,9	1,3 ± 0,5
29	PtdCho	20:4, 22:6	0,0	1,2 ± 0,0	nd	nd
30	PtdCho	20:4, 22:5	0,0	1,0 ± 0,1	nd	nd

The phosphatidylcholine species description comprises the *sn*-1 linkage type followed by the radyl chains on the *sn*-1 and *sn*-2 position, respectively. "Total cell" values are mean of three independent experiments. Most abundant species representing over 5 Mol % are marked in bold. Abbreviations: AlkCho, 1-alkyl, 2-acyl phosphatidylcholine; BSF, bloodstream-form *T. brucei*; EnylCho, 1-alkyl-1-enyl-2-acyl phosphatidylcholine; nd, not detected; PCF, procyclic-form *T. brucei*; PtdCho, diacyl phosphatidylcholine.

## 4. Discussion

The glycosome is a major contributor to kinetoplastid energy metabolism and essential for glycolysis ^3, 4, 47^. Flux rates through the glycolytic pathway are high in trypanosomes and - judging from the peptide counts and protein coverage in our analysis - the enzymes are also abundant in *Leishmania*. A model of trypanosome glycolysis that assumes free exchange of glucose between the organelle and the cytosol mirrors the *in vivo* kinetics ^3, 47^. This suggests that the protein responsible for glucose transport should be very active and probably also abundant. Our analysis of the glycosomal membrane proteome, however, failed to identify abundant membrane proteins that might fulfil such a role. Given the large number of proteins that we identified - including multiple membrane proteins from other compartments - it seems unlikely that our failure to detect transporters for such major metabolites could be due solely to lack of sensitivity. Dual subcellular locations could be a possible explanation for some proteins, as previously shown for MCP6 ^17^. The five additional mitochondrial carrier proteins that we detected are predominantly in the mitochondrion of procyclic *T. brucei* ^48^, but the presence of a minor amount in glycosomes cannot be ruled out. If so, they could possibly function as putative glycosomal isocitrate/2-ketoglutarate and fumarate/malate shuttles.

We investigated the lipid composition of glycosomal membranes in *T. brucei* by analysis of the species composition of phosphatidylcholine, the most abundant phospholipid class in membranes of both procyclic and bloodstream form *T. brucei* ^46^. The phospholipid composition of peroxisomal membranes has been investigated in peroxisomes isolated from rat liver and from the yeasts *Saccharomyces cerevisiae* and *Pichia pastoris* ^49– 51^. In rat liver peroxisomes the phospholipid classes and their fatty acid composition were similar to those of homogenates and microsomes, although exposure to the endotoxin lipopolysaccharide (LPS) induced significant changes in phospholipid species composition: in particular, the abundance of both long chain fatty acids (>20 C atoms) and poly-unsaturated fatty acids increased in peroxisomal membranes ^49^. The lipid composition of peroxisomes in yeasts was shown to be rather flexible, predominantly depending on the type and amount of fatty acid supply in the medium ^50, 51^. The lipid composition of glycosomes in Trypanosomatidae has not been investigated before and our results showed that the phosphatidylcholine species composition in glycosomal membranes resembled that of other cellular membranes in both bloodstream-form and procyclic-form *T. brucei.* These results suggest that the lipid composition, and thus the biophysical properties, of the glycosomal membrane is similar to that of the other membranes in trypanosomes. Because of this similarity to other cellular membranes, glycosomal membranes are expected to be also impermeable to small hydrophilic molecules unless transport is facilitated by specialized membrane proteins.

There is accumulating evidence that small solutes enter microbodies through pores ^5, 20, 52, 53^. Evidence from a mammalian Pxmp2 (PMP22) knock-out mouse suggested loss of peroxisomal pores for solutes of under 300 Da ^53^ and this type of function was confirmed when the protein was expressed in insect cells. No Pxmp2 homologue appears to exist in kinetoplastids. Instead, it has been speculated that PEX11 family proteins might contribute to glycosomal porin activity ^5, 8^. Our failure to find abundant novel glycosomal transporters is consistent with the hypothesis that the PEX11 family proteins are indeed responsible for the transfer of small solutes in and out of the glycosome.
