# Supplementary material for: Proteins and lipids of glycosomal membranes from Leishmania tarentolae and Trypanosoma brucei
Source: F1000Res. 2013 Jan 29;2:27. [Version 1] doi: 10.12688/f1000research.2-27.v1 (PMC3814921; doi:10.12688/f1000research.2-27.v1)
Supplement: Peer review history

# Peer review: Referee response for version 1

**Published:** 2013-03-27

**Authors:** Peter Myler

**Affiliations:** 1 Seattle Biomedical Research Institute, Seattle, WA, USA

In this manuscript, Colasante *et al*. describe an exhaustive analysis of the membrane proteins and lipids present in glycosomes from the kinetoplastid protozoan parasite, *Leishmania tarentolae*, and conclude that, in the absence of obvious transporters for the major substrates and intermediates of energy metabolism, these molecules must be transported across the glycosomal membrane via pores formed by known glycosomal membrane proteins, which are orthologues of the peroxisomal membrane proteins PEX11, GIM5A/B, PXMP4, PEX2 and PEX16. 

The paper is well-written and the technical approach is appropriate. While one must be mindful of the adage “absence of evidence is not evidence of absence”, the comprehensive nature of the proteomic analysis in the present study provides a persuasive argument (albeit, not definitive proof) for this conclusion. As a minor point, it could perhaps have been helpful to have indicated the identity of the proteins enriched in the glycosomal membrane fraction (urea pellet) of Table 1B. It would also be good to indicate what proportions of the total glycosomal fraction are represented by the two lanes in this figure.

I have read this submission. I believe that I have an appropriate level of expertise to confirm that it is of an acceptable scientific standard.

# Peer review: Referee response for version 1

**Published:** 2013-02-08

**Authors:** Ralf Erdmann

**Affiliations:** 1 Department of Biochemistry Systems, Institute of Physiological Chemistry, Ruhr-Universität Bochum, Bochum, Germany

Glycosomes are evolutionarily and functionally related to peroxisomes. To fulfill their metabolic functions, the organelles communicate with the cytosol by an exchange of metabolites/products. Driven by the fact that the knowledge on metabolite transport across the glycosomal membrane and the nature of involved protein in this process is still scarce, the authors performed a proteomic approach with purified organelles. 

The paper is well written, the experimental design and results are conclusive. 

Some minor comments should be addressed:

- Figure 1A: Please indicate top/bottom fractions and provide some information of the density at least of the peak fractions. Please mention in the legend how much of the total fractions was subjected to the gel.- Figure 1B: Please indicate the load. Are the membranes enriched compared to the glycosomal fraction? Please also provide more details on the experimental approach in the methods section, like volumes used for resuspension, fractions loaded on the gel, etc.

I have read this submission. I believe that I have an appropriate level of expertise to confirm that it is of an acceptable scientific standard.
